# Current Trends on Solid Dispersions: Past, Present, and Future

**DOI:** 10.1155/2022/5916013

**Published:** 2022-10-22

**Authors:** Ruba Malkawi, Walla I. Malkawi, Yahia Al-Mahmoud, Jawad Tawalbeh

**Affiliations:** ^1^School of Pharmacy, Jadara University, P.O. Box 733, Irbid 21110, Jordan; ^2^School of Pharmacy, Iowa State University, Ames, IA 50011, USA; ^3^School of Business, Teesside University, Campus Heart, Southfield Rd, Middlesbrough TS1 3BX, UK

## Abstract

Solid dispersions have achieved significant interest as an effective means of enhancing the dissolution rate and thus the bioavailability of a range of weakly water-soluble drugs. Solid dispersions of weakly water-soluble drugs with water-soluble carriers have lowered the frequency of these problems and improved dissolution. Solid dispersion is a solubilization technology emphasizing mainly on, drug-polymer two-component systems in which drug dispersion and its stabilization is the key to formulation development. Therefore, this technology is recognized as an exceptionally useful means of improving the dissolution properties of poorly water-soluble drugs and in the latest years, a big deal of understanding has been accumulated about solid dispersion, however, their commercial application is limited. In this review article, emphasis is placed on solubility, BCS classification, and carriers. Moreover, this article presents the diverse preparation techniques for solid dispersion and gathers some of the recent technological transfers. The different types of solid dispersions based on the carrier used and molecular arrangement were underlined. Additionally, it summarizes the mechanisms, the methods of preparing solid dispersions, and the marketed drugs that are available using solid dispersion approaches.

## 1. Introduction

The oral route is the most convenience route for drug adminstration and favore mode of delivery [1]. From the patient's standpoint, swallowing and medication is a comfortable and familiar method of taking medication. As a result, orally delivered drugs are often more effective than alternative modes of administration, such as parenteral, in terms of patient compliance and drug treatment. When an active substance is given orally, it must first dissolve in the stomach and/or intestinal fluids before it can pass through the GI tract's membranes and reach systemic circulation [2]. Therefore, water solubility and/or membrane permeability of the drug molecule are significant contributors to drug absorption from the gastrointestinal (GI) tract, which causes low medication bioavailability of the medications. Consequently, a drug with weak aqueous solubility usually shows a dissolution rate of limited absorption, while a drug with weak membrane permeability usually shows a permeation rate of limited absorption [3].

Pharmaceutical scientists have two approaches to improving the oral bioavailability of pharmacologically active agents: (i) improving the solubility and dissolution rate of poorly water-soluble medications, and (ii) improving the permeability of poorly permeable drugs [4].

In the pharmaceutical literature, a variety of strategies have been used to improve the dissolving capabilities of weakly water-soluble medications other than solid dispersions. Some of these strategies are salt creation, complexation with cyclodextrins, solubilization of pharmaceuticals in solvent(s), and particle size reduction; however, each of these procedures has significant limitations, such as poor yield, expensive, time consuming, and very low drug solubility [5]. On the other hand, formulating pharmaceuticals as solid dispersions provides several processing and excipient alternatives, allowing for greater flexibility for formulating oral delivery systems of poorly tolerated water-soluble medications [6].

Much of the research that has been published on solid dispersion technologies includes medications that are poorly water-soluble and highly permeable to biological membranes as with these drugs dissolution is the rate-limiting step to absorption. As a result, the rate *of in vivo* absorption will be enhanced in tandem with an increase in the drug dissolution rate. Medications having limited water solubility and strong membrane permeability are classified as class II drugs in the biopharmaceutical classification system (BCS). As a result, solid disperion technology has many promises to enhance the oral absorption of BCS Class II drugs and thier bioavailability [7].

The weak solubility of many discovered drugs is a barrier to their possible therapeutic activity. According to statistical reports, it has been estimated that 40 per cent of the novel chemical entities identified today are water-insoluble [8]. Unfortunately, many of these prospective medications are abandoned in the early phases of development due to solubility difficulties. As a result, it is becoming increasingly crucial to identify new ways to overcome solubility limits so that the potential therapeutic benefits of these active compounds can be realized [9].

Solid dispersion formulation is one of the most promising and practical approaches for increasing solubility. According to Chiou and Riegelman [3], solid dispersion systems are “the solid-state dispersion of one or more active substances in an inert carrier or matrix generated by the fusion, solvent evaporation, or melting-solvent process.” Matrix is hydrophilic, whereas the medication is hydrophobic. Simple eutectic mixtures, solid solutions, glass solutions, and glass suspensions, amorphous precipitation in a crystalline carrier, compound, or complicated forms are solid dispersion types [3].

## 2. Solubility

At a particular temperature and pressure, the solubility of a substance is the amount that has entered the solution when an equilibrium is reached between the solution and the excess, that is, an undissolved substance. The dissolved substance is referred to as the “solute,” and the dissolving fluid in which the solute is dissolved is referred to as the solvent, and the two together are referred to as the solution [10].  Table 1 lists the definitions of various solubility terms.

### 2.1. Biopharmaceutical Classification System (BCS)

The biopharmaceutical classification system was first devised in 1995 by Amidon and his co-workers [7]. According to the biopharmaceutical classification system, drug substances can be classified as shown in Table 2.

Bioavailability may be improved by enhancing the solubility and dissolving rate of the class II medication in the gastrointestinal fluids. Medication release is a critical and limiting step for oral drug bioavailability, especially for medicines with limited gastrointestinal solubility. Optimizing the drug release profile of these drugs makes it feasible to improve their bioavailability and reduce side effects [11, 12].

The World Health Organization's (WHO) model list of essential medicines has assigned a biopharmaceutical classification system categorization based on publicly available data. Unfortunately, only 61 of the 130 orally given medications on the WHO list could be classified with accuracy. Eighty-four percent of these medications are classified as class I, seventeen percent as class II, forty-nine percent as class III, and ten percent as class IV [10].

### 2.2. Mechanisms Involved in Enhancing Drug Solubilization by Solid Dispersion Technique

Although the mechanism is not well understood yet, the basic principle includes the complete removal of drug crystallinity and molecular dispersion of the poorly soluble compound in a hydrophilic polymeric carrier [13]. When the solid dispersion is exposed to the aqueous media, the carrier dissolves and the drug is released as fine colloidal particles. This increases the surface area of the dissolution rate and hence the bioavailability of poorly water-soluble drugs. The drug is a soluble hydrophilic carrier and has a better dissolution rate due to the reduction of the particle size and the increase of the particle porosity. The potential advantage of this technique is enormous. Recently, surfactants were included to improve formulations, as in many cases. However, thermodynamic instability and recrystallization of the drug became a problem. Hence, surfactants are used to avoid recrystallization and to potentiate their solubility [14].

### 2.3. The Justification behind the Use of the Solid Dispersion Technique in the Pharmaceutical Industry

The primary purposes of using this technique in pharmaceuticals are [15]

- Enhancing drug solubility
- Enhancing drug stability
- Masking the bitter taste of drugs
- Gaining the desired release profile.

### 2.4. Types of Solid Dispersions

**Figure 1:**
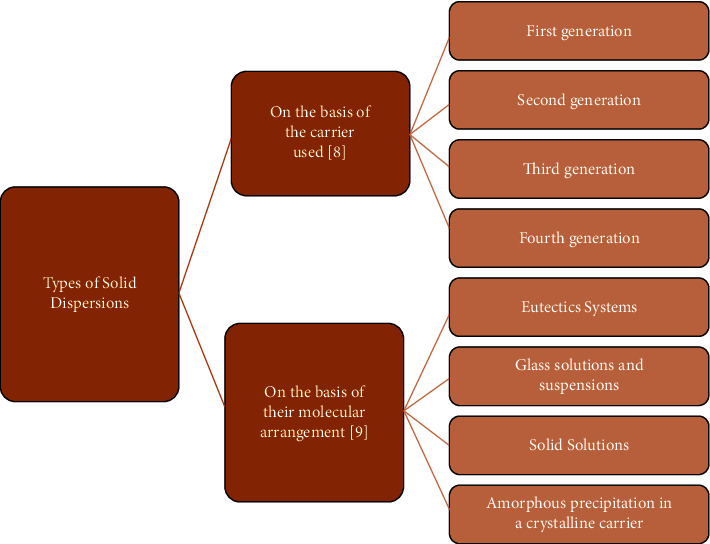
Schematic representation of the solid dispersion types [15].

Figure 1 summarizes the different types of solid dispersion.

### 2.5. Based on the Carrier Used

A carrier must meet the following criteria to be appropriate for enhancing the dissolution rate of a drug. Materials used as carriers are given in Table 3.

- freely water-soluble with intrinsic quick-dissolving capabilities
- nontoxic and pharmacologically inert
- The melting process must be heat stable and it should have a low melting point
- It must be soluble in a wide range of solvents
- It should be able to preferably increase the aqueous solubility of the drug
- Ideally, it should be able to boost the medication's water solubility and be chemically compatible with the drug and should not form a firmly bound complex with it [17]

Based on the carrier used, solid dispersions can be classified into the following four generations [18]:

  First generation: Solid dispersions were formed as the first carriers to be applied in solid dispersions [19]. In this generation, crystalline carriers are used such as sugars and urea. The disadvatange of the first generation is the presence of crystalline nature of the carrier. In which they are thermodynamically stable, and the drug will not be released as fast as the amorphous form [20].
  Second generation: This generation involves the use of amorphous carriers which are usually polymers [21]. These polymers could be synthetic such as polyethene glycols (PEG), povidone, polyvinyl pyrrolidine, and polymethacrylates or natural-based polymers, such as ethyl cellulose, hydroxypropyl methylcellulose (HPMC), and starch derivatives such as cyclodextrins or hydroxypropyl cellulose [22].
  Third generation: It has been proved that the dissolution profile can be enhanced by using a carrier with surface active agent properties. As a result, the use of surface-active agents such as poloxamer 407, compritol 888, ATO, inutec SP1, gelucire 44/14, and inulin as carriers was revealed to be effective in achieving a high purity level of the polymorphic and for increasing *in vivo* bioavailability [23].
  Fourth generation: This type of dispersion is described as controlled release solid dispersion. It contains poorly water-soluble drugs with a short biological half-life. The carriers used are either water-soluble carriers or insoluble water carriers. Solubility enhancement and extended drug release in a controlled manner are the two targets in controlled-release solid dispersion. The water-soluble carriers used in controlled-release solid dispersion include ethyl cellulose, Eudragit, Hydroxypropyl cellulose, and others [24].

### 2.6. Based on their Molecular Arrangement

**Figure 2:**
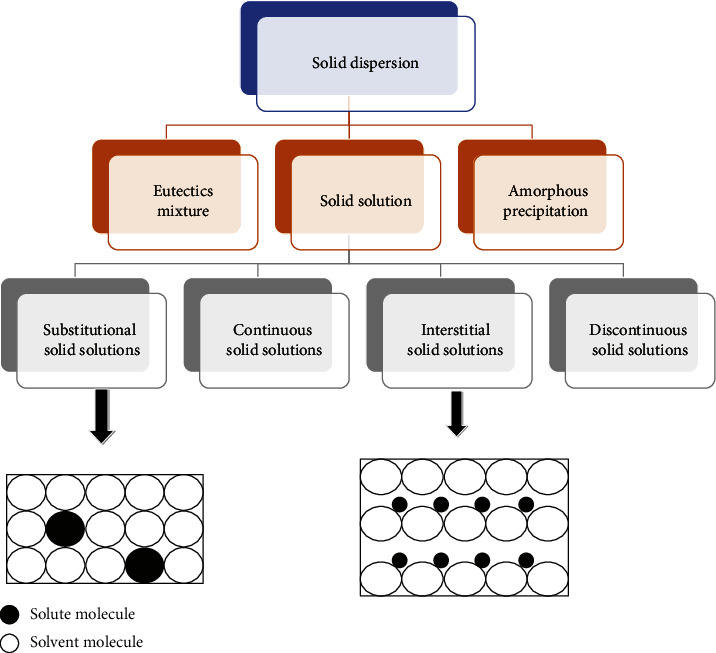
Classifications of solid dispersion [25].

Solid dispersions can be categorized into the following types (Figure 2):

#### 2.6.1. Eutectics Systems

This mixture composes of two compounds in the liquid state that are completely miscible but in the solid state only to a very limited extent. It is prepared through fast solidification of the fused melt of the two compounds, giving a complete liquid miscible product and very little solid-solid solubility. Such a system is thermodynamically intimately mixed with the physical mixture of its two crystalline compounds [26].

#### 2.6.2. Glass Solution and Suspensions

Glass solution refers to the homogeneous glassy system in which a solute is dissolved in a glass carrier, whereas the glass suspensions, in which the precipitated particles are present, are suspended in glass solvent. The lattice energy in such systems is low, and the melting point is not sharp, examples of carriers that form glass solutions and suspensions are urea, citric acid, polyethene glycol, polyvinyl pyrrolidine, and sugars such as dextrose, sucrose, and galactose [26].

#### 2.6.3. Solid Solution

In this system, when the two components crystallize together, they form a single homogeneous phase system. The drug particle size is decreased to its molecular size in the solid solution. As a result, a faster rate of dissolution will be achieved in the solid solution than in the corresponding eutectic mixture. The solution can be categorized (as continuous or discontinuous) depending on the level of miscibility of the two compounds or how the solvate molecules are circulated (substitutional, interstitial, or amorphous) [26].

- Continuous solid solutions:
- The components are miscible in all proportions in a continuous solid solution. Hypothetically, this indicates that the bonding strength between the two components is greater than the bonding strength between the molecules of each individual component. However, solid solutions of this type have not been reported in the pharmaceutical world to date [27].
- Discontinuous solid solutions:
- In the case of discontinuous solid solutions, the solubility of each component in the other component is limited [27].
- Substitutional solid solutions:
- This type of solid solution occurs only if the size of the solute molecules is variable by less than 15% or so from the solvent particles [28].
- Interstitial solid solutions:
- In interstitial solid solutions, the soluble particles fill the interstitial gaps between the solvent molecules in the crystal lattice. Therefore, the solute molecule diameter should be less than 0.59 times that of the solvent molecular diameter [28].

### 2.7. Amorphous Precipitation in the Crystalline Matrix

In the crystalline carrier, the drug may also precipitate in an amorphous form instead of simultaneous crystallization of the drug and the carrier (eutectic system). High dissolution rates are usually produced in this form because of the high energy of the drug in the amorphous state [25].

## 3. Method of Preparation

**Figure 3:**
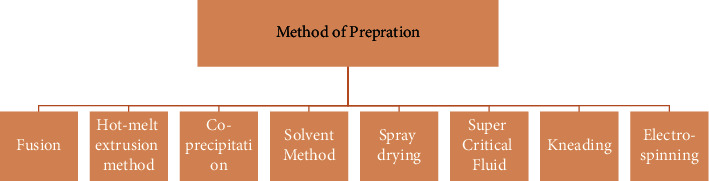
Various methods of solid dispersion preparation [11].

Several techniques for preparing solid dispersion are listed in Figure 3. Generally speaking, there is no best method in solid dispersion to enhance poorly water-soluble drugs. It depends on factors such as the hydrophilicity-hydrophobicity balance of the drug, drug dose, and drug molecular weight. Therefore, trial and error is the best approach the check the proper method that could enhance the drug solubility.

### 3.1. Fusion

Sekiguchi and Obi proposed the fusion method in 1961, also known as the melt method. A physical mixture of drug and polymer is heated to generate a molten mixture, which is then cooled and hardened while vigorous stirring is performed. To reach the desired particle size, the solid mass is crushed, pulverized, and sieved. Despite its popularity, several drawbacks to employing this process in making solid dispersions are present. These drawbacks include a lack of drug-polymer miscibility at the heating temperature. However, surfactants may be used to avoid this issue. Additionally, medications and polymers must be thermally stable at melting temperatures, therefore lower production temperatures are desirable. In addition, the fused mixture must be resistant to recrystallization and phase separation [11].  Table 4 shows examples of decent case studies for the preparation of solid dispersion using the fusion method.

### 3.2. Hot-Melt Extrusion Method

The hot-melt extrusion method is the modern version of the fusion method in which the extruder induces intense mixing of the components. Compared with the traditional fusion method, melt extrusion offers the potential to shape the molten drug-polymer mixture into implants, pellets, or oral dosage forms [4]. However, this method requires the complete miscibility of the drug and the polymer in the molten state. Solubility parameter phase diagrams can be used to predict miscibility and to rationally select the compatible polymer [11].

This process has various advantages, which includes the following [11]:

1. Fewer processing steps because the components are not compressed and the product is not dried, making this procedure simple, continuous, and efficient.
2. Entire mixing at a high shear rate and temperature disaggregates the particles, resulting in a uniform distribution of tiny drug particles in the polymer matrix and molecular level dispersion.
3. In addition, unlike the classical fusion approach, this technique allows for continuous manufacturing, making it appropriate for large-scale production. HPMC, HPMCAS, PVP, PVP, vinyl acetate, and polyethylene oxide are some of the most often utilized polymeric materials in hot-melt extrusion [4].

Over the last decade, hot-melt extrusion (HME) has developed as an effective technique for drug delivery and has started to host such molecules previously considered unviable as drugs. Hot-melt extrusion is an efficient technology for creating solid molecular dispersions and has been proven to produce sustained, modified, and targeted drug delivery after improved drug bioavailability [34]. Nonsteroidal anti-inflammatory drugs (NSAID) and paracetamol were prepared as orally disintegrating tablets using the hot-melt extrusion method [35]. Paracetamol was prepared using the hot-melt extrusion method through granulation paracetamol and filler excipients with different low molecular weight polyethylene glycol using the hot-melt extrusion process. The granules achieved were then mixed with disintegrants and lubricant and were compacted into tablets. The HME granules showed an enhanced drug release profile as compared to the original tablets. More than 80% of the drug was released by tablets that contained 15% of polyethylene glycol within 30 minutes [36], which is the needed amount for paracetamol tablets in the USP 30.

### 3.3. Coprecipitation Method (Coevaporate)

**Figure 4:**
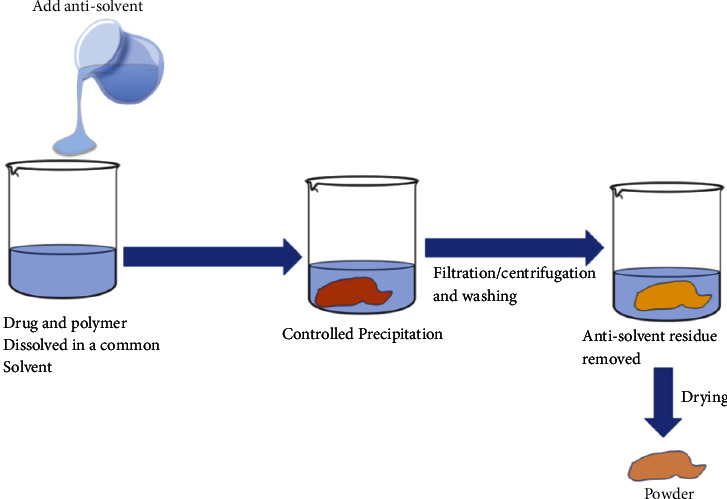
Schematic presentation coprecipitation process [37].

The carrier is accurately weighed and dissolved in water, while the medication is dissolved in an organic solvent. The aqueous carrier solution is then added to the organic drug solution after complete dissolution. After that, the solvents are ejected. The dispersion is crushed, sieved, and dried using a pestle and mortar [37].  Figure 4 shows a demonstration of the process.  Table 5 shows examples of decent case studies for the preparation of solid dispersion using the fusion method.

Ibuprofen is one of the examples of medication that undergoes solubility enhancement using a coprecipitation process. The solubility and the dissolution rate were improved by two-fold and one-fold, respectively.

### 3.4. Solvent Method

The solvent approach entails dissolving both the medication and the polymer in a single solvent and then removing the solvent to create a solid dispersion. This method allows for molecule-level mixing, which is favored for improving product solubility and stability [37].

The fundamental advantage of this approach is that it avoids drug and polymer thermal degradation, which is common when organic solvents are evaporated at low temperatures. When utilizing this strategy, however, formulation scientists face two obstacles [48]. The first issue is to combine the medication and the polymer in a single solvent, which can be challenging if the polarity differences are large. Surfactants are sometimes developed to facilitate medication or polymer solubility in certain solvents. However, their amount in the final dosage form is frequently large, reducing drug loading capacity and potentially causing issues if they are not well tolerated in the body. In addition, this method is expensive due to the necessity to evaporate a substantial amount of the solvent [40]. The second issue is phase separation, which can occur when the solvent is removed. The solution is usually dried by vacuum drying. A rotary evaporator is sometimes used to accomplish rapid drying. The use of higher drying temperatures reduces the time for phase separation. The high molecular mobility of medicine and polymers at high temperatures may speed phase separation [37].  Table 5 shows examples of decent case studies for the preparation of solid dispersion using the solvent evaporation method.

One study was performed with furosemide as it had limited bioavailability, poor solubility, and permeability. The research study intended to assess coprecipitation, kneading, and solvent evaporation by solubility and dissolution enhancement methods. All the approaches were found to enhance the solubility to some extent; however solvent evaporation gave the best results. However, the following order was observed; solvent evaporation > kneading > physical mixtures > coprecipitation [49].

### 3.5. Spray Drying

Spray drying has become a prominent processing method for creating solid drug dispersions. It is used to turn a liquid or a suspension into a dry powder in one step. This method allows for more precise control of process factors, resulting in powders with the required size, shape, density, flow characteristics, and crystalline forms [50]. In spray drying, the solvent evaporates at a rapid rate, resulting in a dramatic increase in viscosity and trapping of drug molecules in the polymer matrix. Drugs with limited water solubility can be spray-dried into extremely fine particles if they are soluble in certain spray-drying solvents. However, the chemical properties of the medication influence the nature of the solid particles generated and spray drying can result in amorphous material, crystalline forms, imperfect crystals, or metastable crystals. Indeed, Mahlin and Bergstrom [51] studied various drug compounds and found that developing an amorphous form is more dependent on the chemical composition of the medications than on process variables. However, the stability of the amorphous forms depends on the process variables. Spray drying provides excellent control over powder characteristics, and it has become the most popular solvent-based production process due to lower manufacturing costs, simplicity of scale-up, and continuous batch production.  Table 6 shows a few examples of decent case studies for the preparation of solid dispersion using the spray drying method.

One study used solid dispersion (SD) techniques and modified the locust bean gum (MLBG) as a carrier to enhance lovastatin drug solubility. Solubility and dissolution studies were used, respectively, to examine the effects of polymer concentration and preparation methods on solubility enhancement. According to the solubility study's findings, lovastatin's solubility increased as MLBG concentration increased. It was discovered that the method used for making the solid dispersions affected the dissolution rate of lovastatin. According to dissolution research, among the different ways of preparing solid dispersions, modified solvent evaporation is the most practical and successful method for improving the solubility of weakly water-soluble lovastatin. The kneading method improves the dissolution rate better than that of coprecipitation because it has other trituration influences on the drug. Spray drying improves the dissolution rate of lovastatin due to enhanced wettability of drug particles and a significant decrease in particle size in the spray drying procedure. The explanation for the greater dissolution rate of solvent evaporation in comparison with other solid dispersions could be due to the availability of increased surface area of particles in the suspension [46].

### 3.6. Supercritical Fluid (SCF) Method

Supercritical fluids have both liquid and gas characteristics. Materials exhibit liquid-like solvent characteristics and gas-like viscosity, diffusivity, and thermal conductivity under supercritical conditions. While the solvent properties are advantageous for drug/polymer solubilization, the gas-like properties considerably improve the fluids' mass transport characteristics [55]. This approach is most commonly used with supercritical carbon dioxide (CO_2_) as a drug and polymer solvent or as an antisolvent. The polymer and medicine are dissolved in supercritical CO_2_ and blasted into a low-pressure zone through a nozzle, generating adiabatic CO_2_ expansion and fast cooling. As a result, this approach enables the creation of drug particles with much smaller particle sizes. The rapid expansion of supercritical solutions is the common name for this technology (RESS) [56]. Current supercritical fluid approaches have shown the ability to generate nanoparticulate suspensions of particles with sizes ranging from 5–2000 nm. This process is considered environmentally friendly because it does not require the use of organic solvents and the small amount of residual CO_2_ trapped inside the polymer causes no risk to patients. CO_2_'s propensity to plasticize and swell polymers can also be exploited. However, the limited solubility of most medicinal chemicals in CO_2_ prevents this method from being used in practice. Several supercritical fluid-processing approaches have been developed to address specific parts of these flaws and to increase solubility. These approaches involve precipitation with a compressed antisolvent, supercritical fluid-enhanced dispersion, supercritical antisolvent processes, gas antisolvent recrystallization, and an aerosol supercritical extraction system [57]. The drug solubility in supercritical CO_2_ has a huge effect on the diameter ranges of the particles formulated by the RESS process. This was demonstrated in a study by Kim et al. [58] when they utilized RESS for the formulation of ultrafine drug particles by applying supercritical CO_2_, with no organic solvent. Three different drugs were used (lidocaine, griseofulvin, benzoic acid) with various solubilities in supercritical CO_2_, and orifice disks and capillary tubes were fitted as an expansion apparatus. The drug solubilities in supercritical CO_2_ and the impacts of different operating parameters on the physical characteristics of the particles formulated by the RESS procedure were experimentally studied. The results showed that the average particle diameter reduced with the solubility for all the drug substances and operating conditions. Response surface methodology was utilized for the optimization of the outcomes, and it was shown that the smallest particle size may be achieved at a temperature of 50°C, a pressure of 17.7 MPa, and a spray distance of 10 cm [59].

### 3.7. Kneading Method

In a glass, a mixture of precisely weighed medication and carriers is wetted with a solvent and is thoroughly kneaded for sometime [16]. In the kneading method, the liquid (which may be water or a hydroalcoholic mixture) is added dropwise while the drug and polymers are triturated in a pestle and mortar. This results in the formation of a slurry and the reduction of particle size, which increases bioavailability because of the kneading action. Then, the mixture is dried and placed through the mesh to bring the contents into homogeneity [60]. Satranidazole-cyclodextrin complexes were made. Following the examination of this complexation, it was discovered that there had been a noticeable increase in solubility [61]. In one study, Olmesartan medoxomil inclusion complexes were created using the kneading approach and were introduced as mouth-dissolving tablets. Complexation increased the solubility and the mechanical stability of the tablets as well as their solubility and dissolution [62]. Efavirenz in PVP K-30 was prepared by two methods, that is, kneading and conventional solvent methods. The two formulations were characterized by DSC, FT-IR, SEM, XRD, and dissolution profile.

A higher dissolution rate has been seen in solid dispersions made by the kneading technique [63]. By kneading approach, Patel created etoricoxib-cyclodextrin complexes. For each material, phase solubility studies were performed to design the phase solubility diagram. Inclusion complexes from this approach showed a significant increase in solubility [64]. By adopting the complexation through kneading approach, nimesulide dissolution was improved [65]. In one study, a BCS class II medication was identified called azithromycin using physical characterization and melting point determination. Melting, kneading, and solvent evaporation were used to generate azithromycin's solid dispersions. From the study's findings, it can be determined that the melting and kneading approaches successfully increased the solubility of the azithromycin to the maximum compared to the solvent evaporation method [66].

### 3.8. Electrospinning Method

This technology combines solid dispersion technology with nanotechnology to be used in the polymer industry. This technique exposes a liquid stream of a drug/polymer solution to a voltage between 5 and 30 kV. Fibres of submicron diameter arise when electrical forces exceed the surface tension of the drug/polymer solution at an air contact [67]. The generated fibres can be collected on a screen to make a woven fabric, or they can be gathered on a spinning mandrel as the solvent evaporates. Surface tension, dielectric constant, feeding rate, and electric field strength all influence the fibre diameter. Because it is the simplest and cheapest technology for preparing nanofibers and controlling the release of medicines, it has enormous potential. In the future, this approach could be used to make solid dispersions [16]. The simplicity and low cost of this method make it advantageous. This technique works well for making nanofibers and managing the release of biomedical treatments. By electrospinning, a nanofiber of polyvinyl alcohol (PVA) : ketoprofen (1 : 1, w/w) was created. The dissolution rate of this nanofiber was significantly greater than that of ketoprofen alone (*p* < 0.05). In a different investigation, indomethacin and griseofulvin were combined in an amorphous form using the electrospinning technique and the PVP was the carrier. For eight months, this mixture remained stable in a desiccator [68].

## 4. Solid Dispersion Characterization

In solid dispersions, the medication in the matrix can take on a variety of molecular configurations. The molecular arrangement in solid dispersions has been studied in several ways. However, much work has gone into distinguishing between amorphous and crystalline materials [16]. For this purpose, many approaches exist to detect the amount of crystalline material in the dispersion. The amount of amorphous material in a sample can never be directly measured, but it can be estimated based on the amount of crystalline material present. It should be highlighted that using crystallinity to measure the amount of amorphous drug makes it impossible to distinguish whether the drug is present as amorphous drug particles or as molecularly dispersed molecules.  Table 7 summarizes the different methods applied to characterize solid dispersions [6].

### 4.1. Marketed Products Used the Solid Dispersion Approach

Several drugs are already on the market and have been prepared using the various approaches of solid dispersion [12]. Some of the products are shown in Table 8.

Different approaches can perform solid dispersion. As a result, solid dispersion methods have been extensively used to improve the solubility of poorly water-soluble drugs.  Table 6 shows the Praziquantel drugs that have been studied using different solid dispersion methods with various carriers.

### 4.2. Solid Dispersion in Polymeric Matrices for *In Vitro* Studies

**Figure 5:**
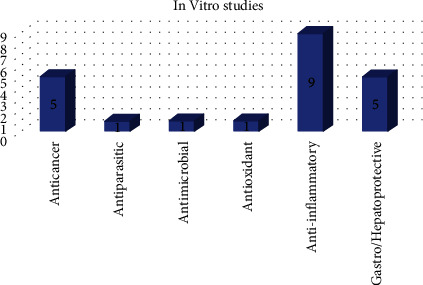
Quantification and classification of *in vitro* studies on solid dispersions published in the period from 2009 to 2020 [78].

This section covers solid dispersions (SDs) used to improve the characteristics and release of poorly soluble natural and synthetic medicines and drug candidates [78]. The synthesis and usage of SDs have been reported in many *in vitro* investigations, which have been numerically quantified and categorized in Figure 5 based on the biological activities of their active compounds, with the major information from this research summarized in Table 9.

### 4.3. *In Vivo* Studies on Solid Dispersions in Polymeric Matrices

**Figure 6:**
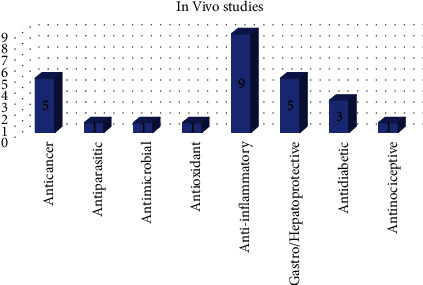
Quantification and classification of *in vivo* studies on solid dispersions published from 2009 to 2020 [78].

As previously indicated, solid dispersions (SDs) have been employed in pharmaceutical technology to overcome some of the limits posed by pharmaceuticals and new bioactive substances, such as the limited solubility and bioavailability [78]. In this regard, as seen in Figure 3 and quantitatively quantified in Figure 6, this section discusses *in vivo* studies on SDs with various biological activities.  Table 10 summarizes the most important aspects of this research.

## 5. Drawbacks of Solid Dispersions

There are several drawbacks that limit the use of solid dispersion in the drug formulation process, including [18] the following:

- Demanding and costly techniques of preparation
- Physicochemical properties reproducibility
- Difficulty merging dosage forms into the formulation
- Scaling up of the manufacturing process
- Stability of medications and solvent

## 6. Conclusion

The oral route of medicine administration is the most common and preferred form of delivery due to its simplicity and convenience of oral administration. From the patient's perspective, ingesting medicine is a convenient and accustomed way to take medicines. As a result, oral medication delivery is frequently more efficient in terms of patient compliance and drug therapy than alternate modes of administration, such as parenteral. When taken orally, an active drug must dissolve in the stomach and/or intestinal fluids before it can cross the GI tract's membranes and enter the bloodstream.

As a result, low medication bioavailability is caused by low drug absorption from the gastrointestinal (GI) tract, which is significantly influenced by the drug's molecule's water solubility and membrane permeability. Solid dispersion systems have proven to be a valuable method for increasing the dissolving properties of poorly water-soluble medications. Solid dispersion technology has gained much knowledge in recent years, but its practical use is still limited. Several ways have recently been tried to overcome limitations and make the preparation more realistic. In addition, the issues involved in incorporating dosage forms into formulation have been increasingly resolved with the development of various solutions. Spraying sugar beads and directly filling capsules are two examples. This study has addressed the aim as well as objectives. This research study was performed by using a review design. There were some major limitations in the study. This research study has no specific methodology section, where the design was specifically described and evidenced. This article has also not talked about any of the processes by which data were collected and the number of articles selected for data collection. These limitations should be addressed in future research studies.

Although there are significant challenges to solve, such as scale-up and production costs, solid dispersion technology has considerable potential for improving the drug release profile of poorly water-soluble medicines.

## Figures and Tables

**Table 1:** The definitions of various solubility terms [9].

Description forms (solubility definition)	Parts of solvent required for one part of solute	Solubility range (mg/ml)	Solubility assigned (mg/ml)
Very soluble	<1	>1000	1000
Freely soluble (FS)	1 to 10	100–1000	100
Soluble	10–30	33–100	33
Sparingly soluble	30–100	10–33	10
Slightly soluble	100–1000	1–10	1
Very slightly soluble	1000–10000	0.1–1	0.1
Practically insoluble	>10000	<0.1	0.01

**Table 2:** Classification of drugs as per the BCS system [7].

Class	Permeability	Solubility
Class I	High	High
Class II	High	Low
Class III	Low	High
Class IV	Low	Low

**Table 3:** Different carriers used in solid dispersion [16].

Category	Carriers
Sugars	Dextrose, sucrose, galactose, sorbitol, maltose, xylitol, mannitol, and lactose
Acids	Citric acid and succinic acid
Polymeric materials	Polyvinyl pyrrolidine (PVP), polyethene glycol (PEG), hydroxypropyl methylcellulose (HPMC), methylcellulose (MC), hydroxyethyl cellulose, cyclodextrin, hydroxypropyl cellulose, pectin, and galactomannan
Insoluble or enteric polymer	Hydroxy propyl methylcellulose phthalate (HPMCP), EudragitL100, Eudragit E100, Eudragit RL, Eudragit RS
Surfactants	Polyoxyethylene stearate, poloxamer 188, deoxycholic acid, tweens, and spans
Miscellaneous	Pentaerythritol, pentaerythrityl tetraacetate, urea, urethane, and hydroxy alkyl xanthine

**Table 4:** Shows examples of decent case studies for the preparation of solid dispersion using the fusion method.

Drug name	Solubility of the pure drug (mg/L) at 25°C	Solubility of the solid dispersed drug at 25°C	Drug release of the pure drug at 37°C after an hour	Drug release percentage of the solid dispersed drug at 37°C after an hour	Polymer used	Reference
Spironolactone	0.02354	0.06173	27.25	74.24	Polyethene glycol 4000	[29]
Carvedilol	0.002	0.012	42.6	93.214	Poloxamer 188	[30]
Cefuroxime axetil	0.412	5.886	10	92	Poloxamer 188	[31]
Luteolin	1.93 × 10^−5^ (at 20°C)	—	13.11	97.78	Polyethylene glycol 4000	[32]
Atorvastatin	<1	—	60	99	Polyethylene glycol 6000	[33]

**Table 5:** Shows examples of decent case studies for the preparation of solid dispersion using the solvent evaporation method.

Drug name	Solubility of the pure drug (mg/L) at 25°C	Solubility of the solid dispersed drug at 25°C	Drug release percentage of the pure drug at 37°C after an hour	Drug release percentage of the solid dispersed drug at 37°C after an hour	Polymer or carrier used	Reference
Nisoldipine	0.005	5	18	75	Polyvinylpyrrolidone	[38]
Nebivolol	0.0403	1.8135	20	98.17	Kleptose HPB, PEG 6000	[39]
Carvedilol	0.002	0.07	7	79	Polyvinylpyrrolidone K 30	[40]
Dutasteride	0.00006	0.0187	60	95.1	PEG 6000	[41]
Cefpodoxime proxetil	0.07211	0.171	20	70	Urea	[42]
Famotidine	0.405	10.436	80 after 35 min	100 after 35 min	Xyloglucan and hyaluronic acid	[43]
Clarithromycin	0.33	—	10	100	Urea	[44]
Ebastine	0.0017	0.014	22	99.68	Avicel® PH101, Avicel® PH 102, croscarmellose sodium(CCS), and sodium starch glycolate (SSG)	[45]
Lovastatin	0.0013	0.00372	30	75	Locust bean gum	[46]
Butein	0.0031	0.114	10 after 24 min	100 after 24	Poloxamer 407 and polyvinyl pyrrolidine K-30	[47]

**Table 6:** Shows examples of decent case studies for the preparation of solid dispersion using the spray drying method.

Drug name	Solubility of the pure drug (mg/L) at 25°C	Solubility of the solid dispersed drug at 25°C	Drug release percentage of the pure drug at 37°C after an hour	Drug release percentage of the solid dispersed drug at 37°C after an hour	Polymer or carrier used	Reference
Celecoxib	0.003–0.007	—	59	100	PEG 6000	[52]
Ritonavir	Practically insoluble in water	0.161	8	100	Hydroxypropyl methylcellulose	[53]
Apigenin	0.002	0.016	23	85	Poloxamer	[54]

**Table 7:** Different characterization methods to assess solid dispersion [6].

Characterization	Technique	Purpose [69]
Drug-carrier interactions	Differential scanning calorimetry (DSC), Fourier transform infrared spectroscopy (FTIR), Raman spectroscopy, and solid-state NMR studies	Used for studying biological reactions and for the validation of production materials and identification of unknown compounds. It is also used for detecting any new interaction

Drug-carrier miscibility	Hot-stage microscopy (HSM), differential scanning calorimeter (DSC), X-ray diffraction (XRD), and nuclear magnetic resonance (NMR)	Used for identifying thermal transition and for achieving a three-dimensional model

Surface properties	Dynamic vapour sorption, inverse gas chromatography, atomic force microscopy, and Raman microscopy	Used for supplying details on particle surface thermodynamic properties, such as surface free energy, acid-base interactions, enthalpy, and entropy. Moreover, it is also used for providing details of the solvent quantity that was absorbed on the sample surface

Physical structures	Scanning electron microscopy, surface area analysis, surface properties, dynamic vapour sorption, inverse gas chromatography, atomic force microscopy, and Raman microscopy	Used for determining the area or pore size of the surfaces sample surface

Stability	Humidity studies, isothermal calorimeter, differential scanning calorimeter (DSC), dynamic vapour sorption, and saturated solubility studies	Used for measuring the reaction between the sample molecules

Amorphous state in solid dispersion	Differential scanning calorimeter (DSC), hot-stage microscopy (HSM), humidity stage microscopy, polarized light optical microscopy, and powder X-ray diffraction	Used for providing a humid environment and for studying the surface properties of the amorphous compounds

Dissolution rate	Dissolution studies, intrinsic dissolution, and dynamic solubility studies	Used for studying the dissolution profile

**Table 8:** Marketed products [70].

Product name	Drug name	Carrier used	Method used	Company name	Reference
Grispeg®	Griseofulvin	PEG	Melt process, the exact process is unknown	Pendinal pharm inc.	[71]
Cesamet®	Nabilone	PVP	Process is unknown	Eli lilly	[71]
Sproranox®	Itraconazole	Hypromellose, HMPC, PEG 20000	Spray layering	Janssen	[71]
Rezulin®	Troglitazone	PVP	Melt-extrusion	Pfizer	[71]
Hepcure®	Hepatitis type b	Amorphous adefovir dipivoxil in solid dispersion	Amorphous adefovir in solid dispersion	CJ CheilJedang	[22]
Keletra®	Lopinavir	PVPV	Melt-extrusion	Abbott	[71]
Afeditab®	Nifedipine	Poloxamer or PVP	Melt/absorb on the carrier	Elan corp, Ireland	[71]
Certican®	Everolimus	HPMC	Melt or spray drying	Novartis, Switzerland	[71]
Fenoglide®	Fenofibrate	PEG	Unknown process	Life cycle pharma, Denmark	[22]
Nivadil®	Nivaldipine	HPC/HPMC	Solvent method	Fujisawa pharmaceuticals co., ltd	[72]
Nimotop®	Nimodipine	PEG	Unknown process	Bayer	[73]
Torcetrapib®	Torcetrapib	HPMC		Pfizer, USA	
Ibuprofen®	Ibuprofen	PEG, HPMC, and PVP	Melt-extrusion	Soliqs, Germany	[71]
Incivek®	Telaprevir	HPMC as	Spray drying	Vertex	[74]
Prograf®	Tacrolimus	HPMC	Wet granulation	Fujisawa pharmaceuticals co., ltd	[74]
Cymbalta®	Duloxetine	HPMC AS	Unknown process	Lilly, USA	
Noxafil®	Posaconazole	HPMC AS	Melt extrusion	Merck	[74]
Intelence®	Etravirine	HPMC	Spray drying	Tibotec, Yardley, PA	[71]
Incivo®		HPMC	Spray drying	Janssen pharmaceutica, Belgium	[74]
Isoptin SRE-240®	Verapamil	Various	Melt-extrusion	Soliqs, Germany	[71]
Isoptin SR-E®	Verapamil	HPC/HPMC HPC/HPMC Abbott	Spray drying	Abbott Laboratories, USA,	[75]
Crestor®	Rosuvastatin	HPMC	Solvent evaporation	AstraZeneca	[76]
Zelboraf®	Vemurafenib	HPMC as	Coprecipitation	Roche	[71]
Zortress®	Everolimus	HPMC	Spray drying	Novartis, Switzerland	[74]
Kalydeco®	Ivacaftor	HPMC as	New solvate of ivacaftor, processes, exact process unknown	Vertex	[77]

PVP: polyvinylpyrrolidone; HPMC: hydroxypropylmethylcellulose; PEG: polyethyleneglycol; HPC: hydroxypropylcellulose; and HMPC AS: hydroxypropylmethylcellulose acetylsuccinate.

**Table 9:** *In vitro* studies on solid dispersion.

Drug name	Carrier used	Method used	Activity	Reference
Niclosamide	OHPP	Spray drying	Anticancer	[79]
Paclitaxel	OHPP	Spray drying	Anticancer	[80]
Paclitaxel	PVP/VA TPGS	Spray drying	Anticancer	[81]
Brij®L4	Chrysin	Solvent evaporation	Anticancer	[82]
Curcumin (CM)	Poloxamer 407	Solvent evaporation	Anticancer enzyme inhibitory/antioxidant anti-inflammatory	[83]
Zn (II)-curcumin complex	PVP K30	Solvent evaporation	Anticancer	[84]
Telaprevir	HPMC, PVP K30, PEG 6000	Kneading	Anticancer	[85]
Angelica gigas nakai	Soluplus®	Hot melting extruder	Anticancer	[86]
Berberine hydrochloride (HB)	Eudragit S-100	Solvent evaporation	Anticancer	[87]
IIIM-290	PVP K30	Scanning electron microscopy	Cytotoxic	[88]
Benznidazole	Poloxamer 407	Solvent evaporation	Antichagasic	[89]
Benznidazole	Low-substituted HPC	Solvent evaporation	Antichagasic	[90]
Ursolic acid	Gelucire 50/13	Solvent evaporation	Antichagasic	[91]
Praziquantel	PVP K30, PVP/VA, kollidon-cl-m, and sodium starch glycolate	Solvent evaporation	Antischistosomal	[92]
Praziquantel	PVP K30	Spray congealing	Antischistosomal	[93]
Artemether	Soluplus, PEG 400, lutrol F127, and lutrol F68	Hot-melt extrudate	Antimalarial	[94]
Lumefantrine	Soluplus, kollidon VA64, and plasdone S630	Hot-melt extrudate	Antimalarial	[95]
Abietic acid	Chitosan	Solvent evaporation	Antimicrobial, antioxidant	[96]
Gatifloxacin	Pluronic F127	Solvent evaporation	Antimicrobial	[97]
Curcumin	PVP K30	Coprecipitation	Antimicrobial	[98]
Curcumin	HPMC	Coprecipitation	Antimicrobial	[99]
Curcumin	Poloxamer 407	Coprecipitation	Antioxidant	[100]
Quercetin	PVP K25	Solvent evaporation	Antioxidant	[101]
Coenzyme Q10	Mannitol	Solvent evaporation	Antioxidant	[102]
Usnic acid	PVP K30	Spray drying	Antioxidant	[103]
Luteolin	PEG 4000	Solvent evaporation, fusion, and microwave irradiation	Antioxidant	[32]
*α*, *β*-amyrin	PVP K30, PEG 6000, and HPMC	Kneading	Anti-inflammatory	[104]
Curcumin	HPMC	Solvent evaporation	Cytoprotective	[105]

OHPP, octenylsuccinate hydroxypropyl phytoglycogen; IC50, half inhibitory concentration; PVP/VA, polyvinylpyrrolidone/vinyl acetate; TPGS, D-*α*-tocopherol polyethylene glycol-1000-succinate; PVP, polyvinylpyrrolidone; SD, solid dispersion; AChE: acetylcholinesterase; BChE, butyrylcholinesterase; CM, curcumin; GST, glutathione S-transferase; MAO, monoamine oxidase; LPS, lipopolysaccharide; NO, nitric oxide; PEG, polyethylene glycol; HPMC, hydroxypropyl methylcellulose; ITG (chloroquine-resistant cell line); DPPH, 2,2-diphenyl-1-picryl-hydrazyl-hydrate; HPMCAS, hydroxypropyl methylcellulose acetate succinate; MIC, minimum inhibitory concentration; ROS, reactive oxygen species; and t-BHP, tert-butylhydroperoxide.

**Table 10:** *In vivo* studies with solid dispersion.

Drug name	Carrier used	Method used	Activity	Reference
IIIM-290	PVP K30	Solvent evaporation	Anticancer	[88]
9-nitrocamptotheci	Soluplus®	Solvent evaporation	Anticancer	[97]
(−)-oleocanthal	(+)-xylitol	Hot melt fusion	Anticancer	[106]
Zinc(II)-curcumin complex	PVP K30	Solvent evaporation	Anticancer	[84]
Selaginella doederleinii hieron	PVP K30	Coprecipitation	Anticancer	[107]
Benznidazole	Low substituted HPC	Solvent evaporation	Antichagasic	[90]
Curcumin (CM)	PVP K30	Coprecipitation	Antimicrobial	[98]
Taurine-zinc complex	PVP K30	Spray drying	Antioxidant	[108]
Triacetylated andrographolide (TA)	Kollidon (VA64)	Solvent evaporation	Anti-inflammatory	[109]
Curcumin	PVP K30, poloxamer 188	Solvent evaporation	Anti-inflammatory	[110]
Aceclofenac	Crospovidone	Solvent evaporation	Anti-inflammatory	[111]
Curcumin	Gelucire®50/13-aerosil®	Hot melt fusion	Anti-inflammatory	[112]
Curcumin	HPMC, lecithin and isomalt	Hot melt extrusion	Anti-inflammatory	[113]
Ibuprofen	PEG 8000	Fusion method	Anti-inflammatory	[114]
Flurbiprofen	Urea and mannitol	Fusion method	Anti-inflammatory	[115]
Meloxicam	Paracetamol	Coprecipitation	Anti-inflammatory	[116]
Chelerythrine (CHE)	PVP K30	Solvent evaporation	Anti-inflammatory	[117]
Curcumin	HPMC	Solvent evaporation	Hepatoprotective	[105]
Nobiletin	HPC	Spray drying	Hepatoprotective	[118]
Silymarin	PVP K30	Solvent evaporation	Hepatoprotective	[119]
Repaglinide	Poloxamer 188	Fusion method	Antihyperglycemic	[120]
Glimepiride	Soluplus1 and PEG 4000	Solvent evaporation	Antidiabetic	[121]
Pioglitazone	PVP K17	Spray drying	Antihyperglycemic	[122]
Hecogenin acetate	HPMC	Kneading	Antinociceptive	[63]

PVP, polyvinylpyrrolidone; SD, solid dispersion; HPC, hydroxypropyl cellulose; CM, curcumin; CM/PVP SD, curcumin/polyvinylpyrrolidone solid dispersion; SOD, superoxide dismutase; PEG, polyethylene glycol; TNF-*α*, tumour necrosis factor alpha; IL, interleukin; NO, nitric oxide; HPMC, hydroxypropyl methylcellulose; and AST, aspartate aminotransferase.

## Data Availability

It is a review of articles with no hyperlinks are applicable.
